# Interprofessional Teams Supporting Care Transitions from Hospital to Community: A Scoping Review

**DOI:** 10.5334/ijic.7623

**Published:** 2024-04-02

**Authors:** Cara L. Brown, Brenda J. Tittlemier, Komal Krishna Tiwari, Hal Loewen

**Affiliations:** 1 Department of Occupational Therapy, College of Rehabilitation Sciences, Rady Faculty of Health Sciences, University of Manitoba, Winnipeg, MB, Canada; 2 Physiotherapy Department, Health Sciences Center, Winnipeg, MB, Canada; 3 University of Manitoba, Winnipeg, MB, Canada; 4 Health Sciences Librarian, Neil John Maclean Health Science Library, Winnipeg, MB, Canada

**Keywords:** care transitions, scoping review, interprofessional teams, discharge planning, older adults

## Abstract

**Introduction:**

Poor outcomes following the transition from hospital back to community living are common, especially for older adults with complex health and social care needs. Some health care systems now have multiple interprofessional teams (in hospital and community) to support care transitions. These teams will need to be well coordinated to improve care transition outcomes.

**Methods:**

We conducted a scoping review to identify and map peer-reviewed literature on how interprofessional teams are working together to support older adults transitioning from hospital back to the community. We used the six-stage framework developed by Levac and colleagues (2010). Procedures were guided by the Joanna Briggs Institute scoping review guidelines.

**Results:**

Our structured search and screening process resulted in 70 articles, published between 2000 and 2022, from 14 counties. Within these articles, 26 programs were described that used interprofessional teams in both the hospital and community.

**Discussion:**

The qualitative articles suggested that effective teamwork is very important for promoting care transition quality, but the quantitative research did not report on team-related outcomes. Quantitative research has described, but not evaluated, strategies for promoting interprofessional collaboration.

**Conclusion:**

Future research should focus on evaluating processes used to promote effective interprofessional teamwork in care transition interventions.

## Introduction

Poor outcomes following the transition from hospital back to community living are common, especially for older adults with complex health and social care needs [1]. Both system and patient-oriented outcomes have been found to be poor, including high rates of hospital readmissions and adverse events, deterioration of physical and mental status, and poor satisfaction with care [2,3,4]. Intervention research on how to improve transitions from hospital to home has had less success for older adults who have complex needs with characteristics such as multiple morbidities, fewer social supports, and/or low socioeconomic status [5,6,7] and as a result, these older adults are often vulnerable to fragmented care [8].

Supporting care transitions requires integrated teamwork of many different health and social care professionals including nurses, pharmacists, physicians, home care coordinators, allied health professionals such as occupational and physical therapists and social workers, and others [8,9,10]. In the hospital, discharge planning is the most typical care transition intervention, where the interprofessional team develops an individualized discharge plan to ensure that the patient’s health and social needs will be met post-hospitalization [9]. In the past, this hospital-based service was the only interprofessional service available to support hospital discharge. However, evidence suggests that interventions to support hospital discharge should extend past the hospital stay to be most effective [3, 11]. Recognizing this gap, some health care systems now offer interprofessional care transition services in the community to provide a variety of health and social care activities such as medication reconciliation, securing equipment needed for independent self-care and fall prevention, and ensuring social needs such as meal and home maintenance and loneliness prevention support is available [12, 13].

The expansion of team-based community and primary care is anticipated to improve the comprehensiveness of care for older adults and reduce institutional care use. However, collaboration and communication between hospital, community and primary care teams is already known to be challenging and to hamper continuity of care [14,15,16]. Studies have documented communication issues between acute and community teams including a lack of, untimely, and/or unreliable communication [14, 15, 17]. Another documented challenge is that when there are many professionals and teams involved in the care of a patient, there can be a lack of accountability because of the shared responsibility for care [14, 15, 17]. Despite these challenges, it is recognized that multiple professional perspectives and having teams to support patients in both the acute and community setting is required to support older adults and their transitions effectively [13, 17]. Effective interprofessional models of care that include strategies to promote within and cross team coordination and collaboration are needed to ensure that the addition of more teams into the health system does not perpetuate fragmentation for the older adult and their family [18].

There are several high-quality reviews on care transitions and/or discharge planning (from hospital to community) in the literature including systematic reviews [1, 3, 6, 13, 19,20,21,22,23] and meta-analyses [8, 24]. However, they had little focus on how interprofessional teams support care transitions across care settings. Rather, they focus on the effectiveness, and/or efficacy of interventions implemented to improve care transitions [3, 8, 13, 19, 20, 23, 25] in relation to pre-discharge interventions [13, 25], hospital-initiated interventions [13, 23], or post-discharge interventions [13] with a primary outcome of hospital readmission rates [3, 8, 13, 19, 20, 23, 24]. Several of the reviews found statistically significant findings in favor of the implemented interventions to reduce hospital readmission rates [3, 8, 19, 20, 23], and yet, poor hospital discharge outcomes continue to be a clinical issue. Straßner and colleagues [11] used eight categories of interventions in their umbrella review of interventions to improve hospital discharge and admissions. One of these categories was teamwork, and their findings were that there were no studies reporting on the effectiveness of teamwork [3, 11].

### Study Aim

There is a gap in our understanding of how teams are working across settings to support care continuity and coordination. This is an important topic since having multiple interprofessional teams in the healthcare system is a new and emerging phenomenon. This scoping review identified the scope of literature reporting on the use of multiple interprofessional teams to support care transitions from hospital to home for older adults, and synthesized the strategies being used by these teams to support care transition coordination.

## Methods

We developed a protocol a priori and published it on Open Science Framework [26]. Our scoping review was informed by the methodological framework developed by Arksey and O’Malley [27] and updated by Levac and colleagues [28]. We also followed the Joanna Briggs Institute Scoping Review guidelines [29] for procedures.

### Stage 1: Identifying the Research Question

The research questions were: a) what are the characteristics of the articles, including publication volume, yearly distribution, type of articles, the setting in which the teams are providing care, and the outcomes being measured?, b) What strategies are interprofessional teams using to support care transition coordination and continuity from hospital to home?

### Stage 2: Identifying Relevant Studies

Our search strategy was developed in collaboration with a health sciences research librarian (HL) using a combination of MESH headings and free text terms. The search strategy was peer reviewed by a second health sciences research librarian. The bibliographic databases: MEDLINE, CINAHL, Cochrane, JBI, EMBASE, and Scopus were searched between January 2000 and July 2022. Consistent with a scoping review method, all study designs with one or more interprofessional teams that support a discharge across the continuum of care were included. Only articles published in English were included in the search as this was the first language of all reviewers. See Appendix A for the detailed search strategy.

### Stage 3: Study Selection

Screening was conducted in Rayyan, a free web-tool for knowledge synthesis projects [30], and COVIDENCE, a web-based collaboration software platform for the updated search review [31]. Screening was conducted in three stages: 1) title screening, 2) abstract screening, 3) full-text screening. Title screening started with three independent reviewers (CB, BT and KT) and discrepancies resolved with discussion. Inclusion criteria details were updated in screening the first 200 titles, and all decisions included in an audit trail. Once title screening inter-rater reliability reached 98%, screening continued with one reviewer with regular meetings to discuss titles requiring discussion to make a screening decision. CB did random checks of title screening to ensure consistent application of criteria. Abstract and full-text screening was completed by two independent reviewers who met with the primary investigator (CB) bi-weekly to review screening. Discrepancies were resolved by consensus or by the primary investigator (CB).

Inclusion criteria for all stages of screening was developed according to the JBI Guidelines [29] that suggest the use of the mnemonic PCC (*population, concept, and context*). Our inclusion criteria for all stages were:

1. *Population*: Adults who were older adults (defined as age 65 or older). Based on concerns that there are also adults younger than 65 at risk for poor transition outcomes with similar health and social circumstances and poor transition outcomes (such as adults with multiple chronic conditions), we also included this population in our search. We included articles that had some participants under the age of 65 if the mean age was 65 or over. The adults needed to have had an unplanned admission to a physical health hospital and then discharged to community. We excluded adults that lived in an institution (e.g., long term care facility; nursing home) prior to hospitalization. We also excluded planned admission (e.g., planned hip replacement surgery), psychiatric and palliative populations.
2. *Concept*: We included articles that had at least one interprofessional team, either in hospital or community. An interprofessional team was defined as three or more health professionals of different backgrounds working together. For example, a team of different physician specialists would not meet this criterion.
3. *Context*: The article needed to address the transition from hospital to community/primary care. We excluded articles that addressed only the hospital stay, and those that addressed only the post-hospital period, since the phenomenon of interest is the transition between the hospital and the community.

### Stage 4: Charting the Data

We developed an Excel data extraction form a priori. We grouped the articles by type (e.g., commentary; qualitative; quantitative) and tailored the extraction for each article type. The extracted data were on: (1) extent (i.e. year of publication; article type; research design), (2) study scope (i.e., study purpose, patient population, level of outcomes [patient-oriented; system-oriented; team function oriented]), setting, setting type, provider type, country of origin); and, (3) strategies used by teams to support continuity (e.g. interprofessional team organization; clinical and administrative strategies). We pilot tested the extraction form and then one author extracted the data from the studies and entered it into the form, with regular meetings with the principal investigator (CB) to review the extraction process. We used a slightly different process for extracting information on the strategies used by interprofessional teams to support care transitions across settings. For this analysis, we used a narrative synthesis approach, using words and text to summarize the articles [32]. The first author (CB) reviewed the intervention or program description of these articles, extracting words or phrases that described features of the intervention of program that were done by the team(s) to support the patient’s care transition. Some of these features were extracted verbatim while for others, CB applied interpretation to summarize the nature of the feature. For example, if there was detailed description of the role of each of the team members to support the care transition, this was summarized as “role clarity of team members”. We extracted text related to the clinical features and strategies of interprofessional teams (e.g., developing interprofessional care plans) as well as the organizational and administrative strategies (e.g., cross-site clinical pathways). We only extracted text related to the team working together, not text explaining roles of individual team members. We added, combined, and collapsed the text into headings that allowed for synthesis of the data that provides meaningful information on the breadth and depth of strategies included in the body of literature. Thus, the extraction and collation (Stage 5) occurred simultaneously for this portion of the analysis.

### Stage 5: Collating, Summarizing and Reporting the Results

We collated the extracted characteristics using categorization and simple counts, except for the narrative synthesis which was described above.

### Stage 6: Consultation

An informal consultation process was conducted by the principal investigator prior to refining the scoping review question. This process involved discussion with policymakers and public researchers (individuals interested in contributing to research who had lived experience with transitions from hospital to home as a patient themselves or as a caregiver) regarding their priorities in relation to research about transitions from hospital and transitional care. Policymakers were interested in how to evaluate the success of newly developed and implemented transitional care programs from both the system and patient experience perspectives. This discussion informed our scoping review in that we did not exclude any articles based on outcomes measured, we included adult populations at risk as well as older adults in the search strategy, and we developed data to outline the specific strategies being used by teams in the current body of literature to support care transitions.

## Findings

**Figure 1:**
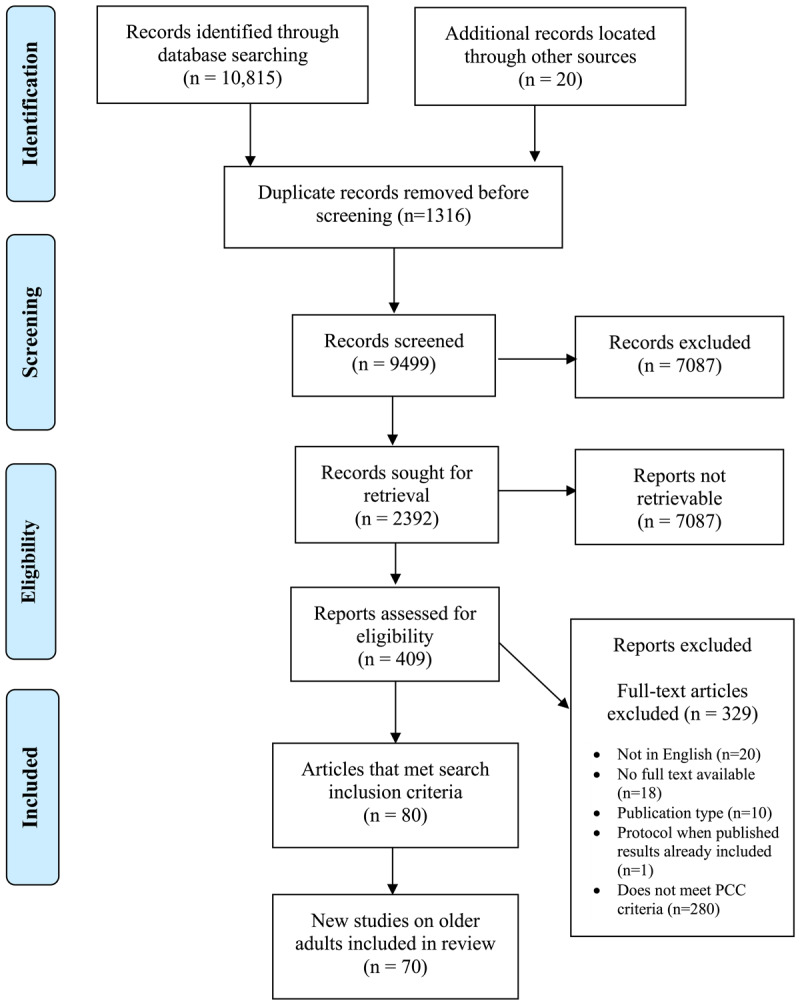
PRISMA diagram.

We conducted an initial search in August of 2020 of articles dating back to January 2000. The search was updated on October 14, 2022, to extend the search from August 2020 to October of 2022. Our initial and updated search resulted in a total of 10,815 records, with an additional 20 records identified through hand searching reference lists of review articles that were included in these records. Title screening resulted in the exclusion of 7,078 articles, and abstract screening another 1,983 articles. We reviewed the full text of 409 articles, and a total of 80 articles met inclusion criteria. However, there were only 10 articles that were not specific to older adults as defined as 65 and over. Most of these articles were about a specific health condition and did not meet our goal of providing insight into adult populations with multiple morbidities that may be under 65 years old. Therefore, we included only the older adult population in this review for ease of interpretation of the findings resulting in a final total of 70 included articles. See Figure 1: PRISMA diagram for screening details.

## Article Characteristics

See Appendix B for the details of all included articles.

### Article Type/Research Design

Of the 70 included articles, there were 32 quantitative research articles (randomized controlled trials [RCT], cohort, quasi-experimental, fidelity of RCT, descriptive), 15 commentaries (including non-structured reviews), eight program descriptions, five qualitative research articles, three protocols, three case studies, two mixed methods studies, one systematic review (narrative), and one theoretical paper. Of the 33 quantitative research studies, 18 were randomized controlled trials.

### Year of Publication

**Figure 2:**
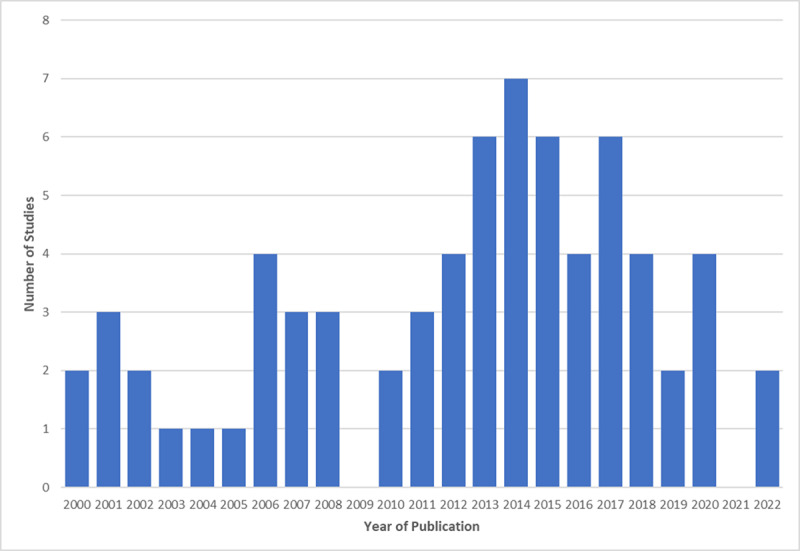
Number of Studies per Year of Publication.

The topic of care transitions and teams grew from the year 2000 and peaked in 2014. See Figure 2.

### Country of Origin

The articles were from 14 different countries. Most of the articles were from countries with developed economies (67 of 70 articles). There were 29 articles from the United States of America, eight from England, eight from Sweden, six from Norway, six from Australia, three from Denmark, two from Singapore, two from Canada, and one each from Argentina, Poland, Scotland, Slovenia, Spain, and Switzerland (total six).

### Characteristics of Programs and Interventions Described in the Literature

#### Transition Setting

This refers to the initial and destination setting of the patients, displayed in Table 1. Most of the articles (n = 48) focused on transitions from an inpatient acute care setting to care provided in the home. However, there is some heterogeneity in relation to setting. Initial settings where the care transition intervention started included the acute care setting, stroke care units, the emergency department and inpatient rehabilitation. Some articles studied the transition from these initial settings to home, and some studied the transition to inpatient transitional care.

**Table 1:** Setting of program or intervention discussed or studied.


INITIAL SETTING	DESTINATION SETTING	NUMBER OF ARTICLES (PERCENTAGE)

inpatient acute care	home	48 (68.6)

emergency department through to inpatient care	home	11 (15.7)

inpatient acute care *or* inpatient rehabilitation	home	3 (4.2)

stroke unit	home	3 (4.3)

inpatient rehabilitation	home	2 (2.9)

acute care	home or inpatient transitional care	2 (2.9)

inpatient acute care	transitional care	1 (1.4)


#### Outcomes

Of 43 articles that reported on quantitative outcomes of a care transitions program or intervention, 27 (62.7%) included or were planning to use patient-oriented outcomes (e.g. self-rated health; symptoms; physical functioning), 29 (67.4%) used or were planning to use health service outcomes (e.g. hospital readmission, length of stay), one provided results related to intervention fidelity and one focused on provider outcomes. We were interested to know if the study that examined provider outcomes looked at how well the interprofessional teams worked together to promote continuity of care. In that study, Preen and colleagues [10] reported on the results of a family physician survey that asked about their satisfaction with the timeliness and quality of hospital communication regarding the hospital discharge plan and the care plan developed by the hospital staff. Since the survey only included the perspective of the family physician, the results of this study provide limited information on how well the interprofessional team was working together.

### Strategies Used by Interprofessional Teams to Support Care Transitions

Here we report on findings from our second research question that asked, what strategies are interprofessional teams using to support care transitions across health care settings? This section first discusses the findings from qualitative articles that collected and reported on data specific to interprofessional teams working to support care transitions from hospital to home. Second, it presents a synthesis of the strategies used by interprofessional teams to promote collaboration and communication across hospital and community boundaries in 26 interventions or programs described in the quantitative literature.

#### Qualitative Findings

The qualitative literature generally supported the use of interprofessional teams for care transition planning, with the primary advantage being that different team members provide different perspectives to the planning [33]. Of all the 80 articles that met the inclusion criteria for this review, only one of them, a qualitative article, looked at how teams support care transitions as a primary objective [34]. In this article, Duner and colleagues [34] report on the professional collaboration and professional boundaries in two interprofessional care planning teams – one hospital team and one community-based team. They found that professionals worked better together during the assessment phase and recommended clarifying the roles of different professionals on the team to improve team working during planning and decision-making phases.

These four articles taken together outline supports and features of well-working teams for best supporting care transitions through interviews with health professionals. An important support for well-working teams is organizational support for taking the time and space needed for meeting as a team and learning about each other’s roles [33, 35]. For example, Baillie and colleagues [35] explored the impact of the organizational integration of a hospital and community area team and found that despite the organizational integration, there continued to poor communication between the acute and community settings because the teams had not been given the opportunity to develop relationships and continued to have little understanding of each other’s roles. This finding was supported by Sims-Gould and colleagues [36] who found that understanding of interprofessional team roles needs to extend beyond their own care setting. Further, organizational support for taking time to develop relationships also supports the development of trust between team members, including trust within site interprofessional teams, and trust between teams in different settings [33,34,35].

Another important feature identified was the ability to blur interdisciplinary boundaries so that team members could support each other’s work [33, 34]. However, there first needs to be role clarity of each other’s roles as well as clear jurisdictional boundaries for decision-making [34]. Another important feature was “open, honest, continuous, and timely” communication [33] (p. 578). More than one study identified the communication between the hospital and the community team as particularly vulnerable to break-down [33, 35]. In addition, both formal and informal communication opportunities were seen to be important [36]. Teams also need to be aware of who is responsible for communication with the patient and family, and having case conferences for complex decision-making were seen to be effective [33].

Finally, care pathways were identified as a helpful tool for interprofessional collaboration to support care transitions but were limited to being useful for those patients who “stay on the pathway”. When the patient was no longer following the standardized pathway, the team again had to contend with challenges with communication and coordination [36]. This relates to the finding by Bull & Roberts [33] that it is essential to have an individual responsible for coordinating efforts – when the care pathway can no longer support coordination, another mechanism needs to be in place.

#### Quantitative Findings

To extract the strategies used by interprofessional teams that support care transitions, we reviewed the 45 articles that represented 35 different interventions of programs (see Appendix C). We were most interested in how interprofessional teams support the transition across the boundary of the hospital to the home since this has been identified as best practice for care transitions [11]. Therefore, we first determined which of these 35 programs or interventions included support from teams in both the initial and final setting, either by providing care with two teams, or by having a bridging intervention. We defined bridging as one or more of the team members providing care outside of the organization with whom they are affiliated to provide patient care continuity across the hospital/community boundary. For example, a hospital-located case manager who provides follow-up home visits and communicates needs to the primary care provider or team. In total, there were 26 programs/ interventions that we included in our analysis of the strategies being used by interprofessional teams to ensure coordination and continuity between and within teams to support care transitions across the hospital/community boundary.

Table 2 presents the organizational and clinical strategies used by interprofessional teams to support care transitions across the hospital boundary. The most common organizational strategies were in using boundary-crossing models of care, ensuring role clarity between health professions and teams, and having consistent meetings. The most common clinical strategies were to use interprofessional assessment and intervention and to have a case manager to coordinate care. Less commonly reported strategies to support well-working teams from an organizational perspective included shared education and training, involvement of the team in program planning and evaluation, and the implementation of strategies to embrace the philosophy of interprofessional teamwork (like working on reducing interprofessional hierarchy in meetings).

**Table 2:** Strategies used by Interprofessional Teams to Support Older Adult Care Transitions.


STRATEGY CATEGORY	SPECIFIC STRATEGIES (REFERENCES REFER TO APPENDIX C)

Organizational and Institutional Strategies and Policy	

Interprofessional Care Models that Cross the Traditional Hospital Boundaries	Early supported discharge.^10,13^ Home-based time limited rehabilitation post hospitalization.^13,16,33^ Post-discharge outpatient transitional clinic.^9,19,36^ Transitional/mobile team- care is provided where the older adult is situated.^10,13^ Hospital in-reach. A community team member visits the hospital pre patient discharge.^1,10^ • Hospital out-reach. A member(s) of the hospital team continues to provide service post-discharge.^2,7,12,16,17,18,26,27,33^ Bridging team member who provides care both in hospital and in community.^2,14,26^ Post-discharge care access. The older adult can access care from the hospital team after discharge.^10,17^

Ensure/Promote Role Clarity	Clear roles/tasks for each team member.^9,10,12,14,16,22,32,36^ Ensure clear understanding of responsibilities of each team.^22,34^

Consistent Meetings	Consistent patient care meetings on daily or weekly basis.^5,7,8,22,32^ Consistent administrative meetings.^22^

Team Involved in Program Planning, Evaluation and Quality Improvement	Team develops the program/intervention.^12,19^ Mechanisms are in place to ensure quality improvement in care delivery.^19,22^

Joint Education and Training	Professional education that promotes interprofessional and/or inter-agency learning.^1,32,34^

Operational/Management Integration	Sharing of activities and duties between hospital and community organizations.^22,35^

Embrace Team Collaboration Principles	Philosophy that community teams are a part of hospital team even though not physically present.^24^ Employ team collaboration strategies in meetings.^8,24^

Clinical Strategies		

Interprofessional Assessment and Intervention	Geriatric comprehensive assessment^5,17,34^ Interprofessional team care planning Hospital care plan.^2,30^ Discharge planning.^22,26^ Post-hospital care plan.^1,5,17^ Home visits Joint home visits pre-discharge with both community and the hospital teams.^4,5,10^ Post-discharge home visit by community team.^5^ Post-hospital team visit by hospital team.^17^ Post-discharge monitoring Phone intervention.^7,19,26^ Remote data monitoring.^8^

Case Manager to Coordinate Care	Community-based.^1,5^ Bridging.^2,7,18,21,26,32,34^

Interprofessional Team Structure that is Tailored to Patient Need	Team is responsible for a specific patient population (e.g., health condition or at-risk of hospital readmission).^7,10,12,19,22,26,32,33,34,36^ Flexible team structure so that each patient has the professions they need on their team.^1,16,22^ Team co-management of patient care by inpatient geriatric team and usual inpatient team.^30^

Clinical Tools Shared Between Hospital and Community Teams	Shared care pathways, protocols and patient educational materials.^1,7,32^ Electronic medical record to promote communication, collaboration, care planning and information sharing.^11,19^

Inter-Agency Care Planning	Team members from more than one setting involved in care planning.^16^ Colleagues of same profession from different settings communicate and collaborate on patient care.^22^ Joint meetings with hospital and community teams for adult care planning.^5,10^

Team has Shared Goals for Patients	All team members focus on patient goals that are important according to evidence.^16^


While all the programs or interventions included both hospital and community components, there were many articles that used strategies where only one team member was supporting the communication between the hospital and community, such as using a case manager, or a team member that provided outreach or in-reach services. In addition, even though we only extracted data from articles that had boundary-crossing components to the program or intervention, many of the strategies reported were focused primarily on either the hospital or community interprofessional team, such as hospital discharge planning or post hospital community assessment. There were fewer strategies for supporting collaboration between both the hospital and community teams.

## Discussion

The objective of our scoping review was to determine the extent of the literature on how interprofessional teams support care transitions for older adults. There has been consensus in the literature for some time that an interprofessional team is an important feature in discharge planning [13]. There also has been recognition that care transition interventions are most effective when the intervention extends past the hospital walls [3]. However, there had not been any reviews to date that focused on the interprofessional team as the unit of analysis, rather than the intervention type. Understanding how teams support care transitions across the care continuum is becoming more important, as more interprofessional teams are being added to transitional, community, primary care, and hospital environments [37]. This scoping review is the first review that we know of that elucidates the body of literature on older adult hospital to home care transitions in relation to the role of the interprofessional team.

Our primary finding is that there is very little literature exploring or evaluating interprofessional teamwork as the unit of analysis for older adult care transitions. Only one qualitative article had explored how interprofessional teamwork supports care transitions as its primary objective [34], and none of the articles in our review looked at teamwork as an outcome. The literature describes interprofessional models of care and strategies for promoting coordination and community across health care boundaries as a part of the intervention but does not examine the construct of teamwork as a confounder. The literature focuses primarily on the intervention activities of the interprofessional teams, such as post-discharge follow-up and then correlates this with health system and patient-oriented outcomes. We know that effective teamwork improves patient outcomes and experiences [38], but that poor team coordination can lead to poor outcomes [39]. The impact of team working is a gap in the literature and needs to be further explored, to determine the confounding effects of team working on these outcomes.

Centeno and Kahveci [38] were the only authors in this review to include information about how effective team process was supported, rather than merely describing the configuration and activities of the team. In their study, they emphasized that all team members were encouraged to participate with an “equal voice” in team meetings, and that processes were reviewed and adjusted as a team [39, 40]. A recent review by Stevens and colleagues [41] emphasized that effective interprofessional teamwork cannot be assumed. They found that the power issues that are common in health care teams due to professional hierarchies can result in poor team collaboration, decision-making and communication. Ensuring high quality continuity and coordination between interprofessional teams is particularly important for the topic of care transitions, since many patients report that they feel “abandoned” following hospital discharge and have difficulty accessing needed services [42].

There is not yet adequate literature to conduct a systematic review on the use of strategies to promote interprofessional team collaboration within and between settings for care transition success. The next step for research in this area is to include measures of team function in care transition intervention studies so that the impact of effective teamwork can be considered alongside patient and system-oriented outcomes. There are many tools that can support the measuring of teamwork and is something that should be added to future research on how teamwork impacts care transition outcomes [43]. Looking at how to support interprofessional teams to work together effectively for care transitions is an area for future research. This research could focus on supporting teams to apply the interprofessional collaboration competencies reflected in the CIHC Interprofessional Competency Framework [44] and by using the Interprofessional Collaborative Relationship Building Model (ICRB) [45] as a tool to build relationships. The CIHC framework promotes effective teamwork through the development of skills in role clarification, conflict resolution, collaborative leadership and interprofessional communication. The ICRB provides more depth into understanding how teams develop relationships that can support effective teamwork and can empower team members to positively impact team functioning.

We encountered a common challenge in synthesizing literature on care transitions which is the heterogeneity of the literature, and a lack of reporting on details of interventions. To support synthesis of the literature on care transitions, future research should involve developing a reporting checklist for care transition research to support reporting consistency. The use of a reporting checklist is currently used for the reporting of patient researcher engagement [46] to support reporting consistency between articles. Adopting this strategy of using a reporting checklist for care transitions and team intervention research could be particularly helpful for ensuring that organizational context is included in the description of interventions being delivered by teams.

A scoping review can provide guidance on where there is an adequate body of literature to conduct systematic reviews. Our review found that there may be an adequate body of literature to conduct systematic reviews on having a team that is tailored to specific diagnostic populations and having a case manager as a part of the team. However, a surprising finding was that the quantity of articles published on interprofessional teams supporting care transitions has decreased in the last eight years. We had anticipated that several factors would have resulted in this body of literature increasing, namely, the growth of interprofessional teams in community and primary care [37, 47], the challenges that health and social health care systems are experiencing with supporting an aging population with chronic disease to live in the community [48], and the focus of health systems to maintain short lengths of stay. The age of many of the articles in this review limits the relevance of conducting systematic reviews on some of the topics that were more common in this review.

A limitation in our search strategy was that we may have excluded some papers in our screening because they did not explicitly describe the members of the interprofessional team. Many papers simply stated that the intervention included an interprofessional team without describing the membership, and these papers were deemed ineligible for our review. Another limitation was that our analysis of the strategies used by interprofessional teams to support care transitions was limited by the high variation between studies in the extent and detail to which they described the care transition intervention. Space limitations in journals may have meant that our included articles may have been missing some details on how teams were supported to work together. Regardless, our analysis provides insight into the extent and depth of how recent research has considered coordination and continuity of interprofessional teams across care boundaries to support care transitions and provides important insight into important future research directions.

## Conclusion

There is a large body of literature that includes interprofessional teams in the care transition intervention, but very few articles focus on how to promote coordination and continuity between these interprofessional teams. Considering the evidence on the importance of well-working teams to improve health outcomes, this is an important area for future research. Implementing and measuring these types of teamwork interventions is an important focus for future research on care transitions from hospital to home. Without a deliberate approach to supporting teamwork across boundaries, the addition of more interprofessional teams to the health care system could potentially increase care fragmentation. We need to develop an understanding of how teams can and should work together, alongside the development of evidence on the most effective interprofessional interventions.

## Additional Files

The additional files for this article can be found as follows:

10.5334/ijic.7623.s1 Appendix A. Search Strategy.

10.5334/ijic.7623.s2 Appendix B. Summary of Included Studies.

10.5334/ijic.7623.s3 Appendix C. Interventions/Programs in the Body of Literature.
