# Supplementary material for: Interprofessional Teams Supporting Care Transitions from Hospital to Community: A Scoping Review
Source: Int J Integr Care. 2024 Apr 2;24(2):1. doi: 10.5334/ijic.7623 (PMC11012160; doi:10.5334/ijic.7623)
Supplement: Appendix A. — Search Strategy. [file ijic-24-2-7623-s1.pdf]

Ovid MEDLINE(R)

1. patient care team/
2. interprofessional relations/
3. ((health or patient or medical) adj5 ("care team*" or "healthcare team*" or "health team*")).tw,kf.
4. ((interdisciplinary or inter-disciplinary or interprofessional or inter-professional or multi-profession* or multiprofession* or multi-discipline) adj5 ("care team*" or "healthcare team*" or "health team*" or team* or relation* or etiquette* or relation*)).tw,kf.
5. or/1-4 [inter-professional team]
6. exp "continuity of patient care"/
7. ((plan or plans or planning or patient or care) adj5 (discharg* or transfer* or exit or exiting or dumping or turfing* or transition*)).tw,kf.
8. ((care or treatment*) adj3 (after or follow-up or "follow up")).tw,kf.
9. ((clinical or nursing or patient) adj3 (handoff* or "hand off*" or handover* or "hand over*" or signout* or "sign out*" or signover*)).tw,kf.
10. or/6-9 [patient discharge]
11. 5 and 10 [inter-professional and discharge]
12. exp hospitals/
13. inpatients/
14. hospital*.tw,kf.
15. (care adj3 (center* or centre* or facilit* or critical or intensive)).tw,kf.
16. (inpatient* or in-patient*).tw,kf.
17. or/12-16 [inpatients]
18. exp Aged/
19. retirement/
20. (age* or elder* or older adult* or senior* or retiree* or retired or retirement or geriatric* or pensioner* or old* person* or old* people*).tw,kf.
21. or/18-20 [**aged]
22. poverty/
23. exp "emigrants and immigrants"/
24. homeless persons/
25. vulnerable populations/
26. working poor/
27. (impoverished or poverty).tw,kw.
28. (indigen* or indigent*).tw,kw.
29. ((vulnerable or underserved or homeless or marginali* or "low* income" or "low-income" or "lower-income" or poor*) adj3 (people* or population* or person* or individual* or group* or communit* or neighbo?rhood* or man or woman or men or women or child or children)).tw,kw.
30. ((low or lower or poor* or depressed) adj3 (socio-economic or socioeconomic)).tw,kw.
31. (alien* or emigrant* or foreigner* or immigrant*).tw,kw.
32. homelessness.tw,kw.
33. (disadvantaged or "sensitive population").tw,kw.
34. (working adj2 poor).tw,kw.
35. or/22-34 [vulnerable population]
36. or/21,35 [aged and vulnerable population]
37. 11 and 17 and 36 [final search]
38. limit 37 to english language
