# Supplementary material for: Interprofessional Teams Supporting Care Transitions from Hospital to Community: A Scoping Review
Source: Int J Integr Care. 2024 Apr 2;24(2):1. doi: 10.5334/ijic.7623 (PMC11012160; doi:10.5334/ijic.7623)
Supplement: Appendix B. — Summary of Included Studies. [file ijic-24-2-7623-s2.pdf]

Appendix: Summary of Included Studies does Liu need to be added to this table?

Autho r(s) (year)	Countr y	Study/Paper Purpose	Study Design	Interve ntion/P rogram name	Population	Measu res	Transiti on Setting of Patient (Initial: Destina tion)	Setting of Interpro fessiona l Care	Article includes team(s) working across setting boundar y?
Articles that describe an intervention or program									
Avlun d et al. (2002)	Denma rk	Examine whether regular interdisciplinary comprehensive home visits after discharge from hospital have an effect.	RCT	Compre hensive follow-up visits	Older adults	Patient -oriente d & Health service s	Acute care hospital: Home	Commu nity team; Hospital team bridging to commun ity	Yes
Karlss on, et al. (2016)	Swede n	Effects of Geriatric Interdisciplinary Home Rehabilitation on walking ability and length of stay for older people with hip fracture compared with conventional geriatric care and rehabilitation.	RCT	Geriatri c Interdis ciplinar y Home Rehabili tation (GIHR)	Older adults	Patient -oriente d	Acute care hospital: Home	Commu nity team; Hospital team	Yes
Berglu nd et al. (2015)	Swede n	Analyse effects of a comprehensive continuum of care on frail older persons' life satisfaction.	RCT	Continu um of Care Interven tion	Older adults	Patient -oriente d	Acute care hospital (ED): Home	Hospital team, Commu nity team bridging	Yes

								into hospital	
Lainscak et al. (2013)	Slovenia	Test whether coordination of discharge from hospital reduces hospitalizations in patients with chronic obstructive pulmonary disease (COPD).	RCT	Discharge coordinator intervention for COPD	Older adults	Patient-oriented & Health services	Acute care hospital: Home	Community team; Hospital team bridging to community	Yes
Eklund et al. (2013)	Sweden	To evaluate the effects of the intervention on functional ability.	RCT	Continuum of Care Intervention	Older adults	Patient-oriented	Acute care hospital (ED): Home	Hospital team, Community team bridging into hospital	Yes
Ebrahimi et al. (2017)	Sweden	Evaluate effects of the Continuum of Care (CC) intervention on self-rated health, experiences of security/safety and symptoms for frail elderly people.	RCT	Continuum of Care Intervention	Older adults	Patient-oriented	Acute care hospital (ED): Home	Hospital team; Community team bridging into hospital	Yes
Preen et al. (2005)	Australia	Determine the effects of a multidisciplinary post-discharge continuance of care intervention of quality of life, discharge satisfaction and hospital length of stay.	RCT	Discharge care planning model	Older adults	Patient-oriented & Provider-oriente	Acute care hospital: Home	Community team bridging to hospital	Yes

						d			
Hofstad et al. (2014)	Norway	Compare Early Supported Discharge (ESD) to usual rehabilitation intervention.	RCT	Early Supported Discharge (ESD)	Older adults	Patient - Oriented	Acute care hospital: Home	Community team; Hospital team bridging to community	Yes
Fjaertoft et al. (2004)	Norway	Compare the effects of an Extended Stroke Unit Service (ESUS) with the effects of an Ordinary Stroke Unit Service (OSUS) on long-term quality of life (QoL).	RCT	Extended Stroke Unit Service (ESUS)	Older adults	Patient - oriented	Stroke unit: Home	Hospital team bridging into community	Yes
Fjaertoft et al. (2003)	Norway	Evaluate the long-term effects of an extended stroke unit service (ESUS), characterized by early supported discharge.	RCT	Extended Stroke Unit Service (ESUS)	Older adults	Patient - oriented & Health services	Stroke unit: Home	Hospital team bridging into community	Yes
Allen et al. (2002)	United States of America	Test effectiveness of comprehensive, interdisciplinary post discharge care management for stroke and TIA patients.	RCT	Post discharge care management model for stroke and TIA	Older adults	Patient - oriented	Acute care hospital or inpatient rehabilitation ward: Home	Hospital team bridging into community	Yes
Indred	Norwa	Evaluate the effects of an	RCT	Extende	Older adults	Patient	Stroke	Hospital	Yes

avik et al. (2000)	y	extended stroke unit service (ESUS).		d stroke unit service (ESUS)		- oriented & Health Services	unit: Home	team bridging into community	
Schapira M, et al (2022)	Argentina	Assess whether geriatric co-management combined with an interdisciplinary transitional care intervention can reduce 30-day hospital readmission rate compared to usual care.	RCT	Geriatric co-management model	Older adults	Health Service	Acute care hospital: Home	Hospital team bridging to community	Yes
Inglis et al. (2006)	Australia	Determine the effects of a nurse-led, multidisciplinary, home-based intervention (HBI) in a old and fragile cohort.	RCT	Home-based intervention (HBI)	Older adults	Health services	Acute care hospital: Home	Community team	No
Torp et al. (2006)	Denmark	Evaluate whether an interdisciplinary stroke team could improve existing discharge planning processes.	RCT	Hospital-supported discharge after stroke	Older adults	Patient-oriented & Health services	Acute care hospital: Home	Hospital team bridging to community	Yes
Garcia-Aymerich et al. (2007)	Spain	Evaluate effectiveness of an integrated care intervention on reduced hospital readmissions in chronic obstructive pulmonary disease (COPD).	RCT	Integrated care (IC) intervention	Older adults	Patient-oriented	Acute care hospital: Home	Community team	No
Low et al. (2017)	Singapore	Evaluate the application of integrated practice units (IPU) concept to the organization of a virtual ward model in reducing	RCT	Modified Virtual Ward	Older adults	Health services	Acute care hospital: Home	Community team; Hospital	Yes

		readmissions of high-risk patients.		Care				team bridging to community	
Wierzchowiecki et al. (2006)	Poland	Determine effectiveness of multidisciplinary care for heart failure patients at one year follow-up.	RCT	Multidisciplinary care for heart failure	Older adults	Patient-oriented & Health services	Acute care hospital: Home	Community team (outpatient clinic)	No
Arbaje et al. (2010)	United States of America	Report on a pilot study of the Geriatric Floating Interdisciplinary Transition Team (Geri-FITT)	Quantitative	Geriatric Floating Interdisciplinary Transition Team (Geri-FITT)	Older adults	Patient-oriented	Acute care hospital: Home	Hospital team	No
Ekelund & Eklund (2015)	Sweden	To evaluate longitudinal effects of the intervention in terms of self-determination in daily life for community-living frail older persons.	Quantitative	Continuum of Care Intervention	Older adults	Patient-oriented	Acute care hospital (ED): Home	Hospital team; Community team bridging into hospital	Yes
Allen et al. (2011)	United States of	Describe the theoretical basis for the After Discharge Management of Low Income	Quantitative	After Discharge	Older adults	Patient-oriente	Acute care hospital:	Community team	Yes

	America	Frail Elderly (AD-LIFE) trial to improve chronic illness management and transitional care.		Management of Low Income Frail Elderly (AD-LIFE) trial		d	Home	bridging into hospital	
Stranges et al. (2015)	United States of America	Evaluate the effectiveness of a multidisciplinary practice model on reducing 30-day all-cause readmissions.	Quantitative	Patient-centred medical home model	Older adults	Health services	Acute care hospital: Home	Community team (outpatient clinic)	No
Ohuabunwa et al. (2013)	United States of America	Examine the effectiveness of a transitional care program in low-income older adults.	Quantitative	Coleman intervention for low income	Older adults	Health services	Acute care hospital: Home	Hospital team bridging into community	Yes
Weintraub et al. (2018)	United States of America	To describe the initial experience with IT enabled care management for ischemic heart disease with an emphasis on readmissions and secondary prevention.	Quantitative	Care Management Information Technology Model	Post coronary artery bypass surgery	Patient-oriented & Health services	Acute care hospital: Home	Community team bridging into hospital	Yes
Farhat et al. (2019)	United States of America	Evaluate the effect of an interdisciplinary transitions of care (TOC) service on readmission rates in a geriatric population.	Quantitative	Interdisciplinary transitions of	Older adults	Health services	Acute care hospital: Home	Community team	Yes

				care service					
Mudge et al. (2014)	Australia	Pilot a multidisciplinary follow-up clinic for frequently admitted patients to address transitional care gaps.	Quantitative	Multidisciplinary follow-up clinic	Older adults	Patient-oriented	Acute care hospital: Home	Community team	No
Kongensgaard et al (2022)	Denmark	Evaluate the association between the involvement of relatives in hospital-based multidisciplinary geriatric home visits within 48 hours of hospitalization and 30-day unplanned readmission rates among frail older patients.	Quantitative	Early home visits by hospital geriatric team	Older adults	Health services	Acute care hospital: Home	Hospital team bridging into community	Yes
Lovelace, et al. (2016)	United States of America	Examine the impact of the McGuire Veterans Administration Medical Center (VAMC) transitional care program (TCP) on health service outcomes.	Quantitative	Veterans Administration Medical Center (VAMC) transitional care program (TCP)	Older adults	Health services	Acute care hospital (ED): Home	Hospital team; Bridging team	Yes
Manley et al (2020)	United States of America	Determine the association of a multidisciplinary medication therapy management (MTM) with 30-day readmission rates.	Quantitative	multidisciplinary medication therapy management	Older adults	Health services	Acute care hospital: Home	Community team	No

				(MTM)					
Garnier et al. (2018)	Switzerland	Evaluate the effectiveness of a multidisciplinary transition plan to reduce early readmission among heart failure patients.	Quantitative	Multidisciplinary transition plan for HF	Older adults	Health services	Acute care hospital (ED): Home	Hospital team bridging into community	Yes
Hoover et al. (2017)	United States of America	Evaluate the effectiveness of a care transitions quality improvement (QI) intervention on self-management and readmission rates in older adults with HF.	Quantitative	Coleman intervention for heart failure	Older adults	Patient-oriented & Health services	Acute care hospital: Home	Hospital team bridging into community	Yes
Ljungberg et al. (2001)	Sweden	Evaluate a bridging rehabilitation program for stroke patients.	Quantitative	Extended stroke unit service (ESUS)	Older adults	Patient-oriented; Health services	Inpatient rehabilitation: Home	Hospital team; Community team bridging into hospital	Yes
Hasson et al. (2012)	Sweden	Evaluate implementation fidelity of a complex care continuum intervention for frail elderly people.	Quantitative	Continuum of Care Intervention	Older adults	Fidelity	Acute care hospital ED): Home	Hospital team; Community team bridging into hospital	Yes
Wright et al. (2007)	United States of Americ	Report on a pilot study of the AD-Life trial and describe a planned RCT.	Program Description	After Discharge Care Manage	Older adults	Patient-oriented &	Acute care hospital: Home	Hospital team bridging into the	Yes

	a			ment of Low-Income Frail Elderly [AD-LIFE]		Health services		community	
Lui & Garwood (2015)	United States of America	Describe a medication reconciliation program within a multidisciplinary geriatrics practice that aimed to facilitate transitions of care. .	Program Description	Multidisciplinary transitions of care team	Older adults	Health services	Acute care hospital (ED): Home	Hospital team; Community team; Bridging team consisting of members of hospital and community team	Yes
Palmer (2018)	United States of America	Describe the Acute Care for Elderly (ACE) model and summarize previous research.	Program Description	Acute Care for Elderly (ACE)	Older adults	n/a	Acute care hospital: Home	Hospital team	No
Mashaw (2014)	United States of America	Improve readmission rates by redesigning the discharge and follow-up process of a hospital in a rural area.	Program Description	Readmission prevention team	Older adults	Health services	Acute care hospital: Home	Hospital team bridging into the community	Yes
Cente	United	Describe a transitional care	Progra	Transiti	Older adults	n/a	Acute	Hospital	Yes

no & Kahveci (2014)	States of America	program that aimed to decrease hospital readmission rates.	m Description	onal care model			care hospital: Home	team; Community team bridging into hospital	
Thorne & Jeffrey (2001)	England	Describe an area-wide stroke rehabilitation strategy.	Program Description	Area-wide stroke strategy	Older adults	Patient-oriented & Health services	Acute care hospital: Home	Hospital team; Community team	Yes
Cameron et al. (2000)	Scotland	Determine the impact of a nurse-led multi-disciplinary team on management of elderly patients with functional problems.	Program Description	Nurse-led multidisciplinary team	Older adults	Health Services	Acute care hospital: Home	Hospital team	No
Cavalier & Sickels (2015)	United States of America	Describe initiative that aimed to decrease readmissions in heart failure patients.	Program description	HF collaborative team	Older adults	Health services	Acute care hospital: Home	Hospital team bridging into the community	Yes
Ornstein et al. (2011)	United States of America	Describe a nurse practitioner (NP)-led transitional care program embedded within an existing home-based primary care (HBPC) program.	Mixed-method	Nurse practitioner transitional care program	Older adults	Health services	Acute care hospital: Home	Hospital team bridging into the community	Yes
Markle-Reid	Canada	Examine the effectiveness of an integrated transitional	Mixed-method	Transitional	Older adults	Patient-	Acute care	Community	No

at al. (2020)		care intervention for community-living older adults with stroke and multimorbidity.		care stroke intervention (TCSI)		oriented, Health services	hospital: Home	team	
Hazelett et al. (2006)	United States of America	Determine whether comprehensive post-hospitalization interdisciplinary care management can be an effective care delivery model to improve outcomes in low-income frail elderly.	Protocol	After Discharge Care Management of Low-Income Frail Elderly [AD-LIFE]	Older adults	Patient-oriented; Health Services	Acute care hospital: Home	Hospital team bridging into the community	Yes
Verweij et al. (2018)	Norway	Investigate if unplanned hospital readmission and mortality can be reduced by the Cardiac Care Bridge transitional care program (CCB program).	Protocol	Cardiac Care Bridge	Older adults	Patient-oriented & Health services	Acute care hospital: Home	Hospital team; Community team bridging into hospital	Yes
Watson et al. (2015)	England	Compare a Community In-Reach and Care Transition (CIRACT) service with Traditional Hospital Based rehabilitation (THB-Rehab) provided to the older person.	Protocol	Community In-Reach and Care Transition (CIRACT)	Older adults	Patient-oriented & Health services	Acute care hospital: Home	Community team bridging into hospital	Yes
**Qualitative Articles**									

Baillie et al. (2014)	England	Investigate the care transitions of frail older people within the context of a healthcare system with vertically integrated acute and community healthcare services.	Qualitative	n/a	Older adults	n/a	Acute care hospital: Home		
Duner (2013)	Sweden	Examine professional collaboration and professional boundaries in interprofessional care planning teams.	Qualitative	n/a	Older adults	n/a	Acute care hospital: Home		
Bull & Roberts (2001)	England	Identify the components of effective discharge planning for elders and factors that impede planning.	Qualitative	n/a	Older adults	n/a	Inpatient rehabilitation: Home		
Sims-Gould et al. (2012)	Canada	Examine the elements that constitute a successful transition across the continuum of care from the perspective of health care providers for hip fracture patients.	Qualitative	n/a	Older adults	n/a	Acute care hospital (ED): Home		
Glasby et al. (2008)	England	Report findings from a national evaluation of intermediate care, focusing on the relationship between hospital services and intermediate care.	Qualitative	n/a	Older adults	n/a	Acute care hospital: Home or inpatient transitional care		
Other									
Takahashi et al. (2020)	United States of Americ	Review common models including the Mayo Clinic model for posthospital transition.	Commentary	Mayo Clinic Care Transiti	Older adults	n/a	Acute care hospital: Home		

	a			ons (MCCT) progra m					
Reinh ard & Lind (2012)	United States of Americ a	Describe selected policy initiatives intended to address the healthcare workforce needs of older persons who experience transitions, and their informal caregivers.	Comme ntary	n/a	Older adults	n/a	Acute care hospital: Home		
Johan sson (2012)	United States of Americ a	Describe the importance of a comprehensive care care plan for care coordination.	Comme ntary	n/a	Older adults	n/a	Acute care hospital: Home		
de Leon et al. (2017)	United States of Americ a	Highlight common pitfalls encountered during the transition period and methods to improve patient care and safety.	Comme ntary	n/a	Older adults	n/a	Acute care hospital: Home		
James (2008)	Englan d	Describe how multidisciplinary teams are providing intermediate care for the older population in Southwark.	Comme ntary	n/a	Older adults	n/a	Acute care hospital: Home		
Low et al. (2014)	Singap ore	Description of the collaboration between two family physician-led transitional home care teams from two restructured hospitals that resulted in safe transfer of care.	Comme ntary	n/a	Older adults	n/a	Acute care hospital: Home		
Lowth ian (2017)	Austral ia	Discussion of how to optimize the care transitions of older adults.	Comme ntary	n/a	Older adults	n/a	Acute care hospital: Home		

Naylor & Sochalski (2010)	United States of America	Describe two projects that identified the essential elements of effective care management interventions for the chronically ill population.	Commentary	n/a	Older adults	n/a	Acute care hospital: Home		
Naylor et al. (2020)	United States of America	Describe how the components of the Transitional Care Model can provide a framework for a holistic care approch to older adults with COVID-19.	Commentary	n/a	Older adults	n/a	Acute care hospital: Home		
Oboh (2016)	England	Review strategies and communication tools that can support transfer of care of frail older people.	Commentary	n/a	Older adults	n/a	Acute care hospital: Home		
Winfield & Burns (2016)	England	Provide commentary on the National Institute for Health and Care Excellence and Social Care Institute for Excellence published guidelines on transition from inpatient setting to community.	Commentary	n/a	Older adults	n/a	Acute care hospital: Home		
Tahan (2007)	United States of America	Describe how case management, through care coordination, can play an important role in ensuring safe and effective care transitions.	Commentary	n/a	Older adults	n/a	Acute care hospital (ED): Home		
Arbaje et al. (2014)	United States of America	To characterize health care professionals' perspectives on successful transitional care of older adults out of the hospital, suggestions for improving transitional care, and Pay-for-performance (P4P) strategies as they relate to transitional care.	Commentary	n/a	Older adults	n/a	Acute care hospital or inpatient rehabilitation		

							ward: Home		
Gray et al. (2008)	Australia	Describe transiton care and evidence supporting its use.	Commentary	n/a	Older adults	n/a	Acute care hospital: Home or inpatient transitional care		
Shaid et al. (2017)	United States of America	Present a case study of how a homebound older adult patient with urinary retention is managed by a patient-centered medical home/transitional care model.	Case study	n/a	Older adults	n/a	Acute care hospital: Home		
Bradway et al. (2013)	United States of America	Urologic and transitional care needs of an elderly, cognitively impaired male during and after an acute hospitalization.	Case study	NA	Older adults	n/a	Acute care hospital (ED): Home		
Figved et al. (2019)	Norway	Illustrate multidisciplinary orthogeriatric treatment using a case study.	Case study	n/a	Older adults	n/a	Acute care hospital or inpatient rehabilitation ward: Home		
Sheikh et	United States	Propose an innovative approach to address transitions across	Theoretical	n/a	Older adults	n/a	Acute care		

al. (2018)	of America	multiple health care settings, derived from best practices models.					hospital: Home		
Allen et al. (2013)	Australia	Synthesise literature on multiprofessional communication between health and social care professionals within transitional care for older people.	Systematic review	n/a	Older adults	n/a	Acute hospital care: Transitional care		
Hennessey & Suter (2011)	United States of America	The experience of health care providers with the Community-based transition model.	Commentary	The Community-Based Transitions Model	Older adults	n/a	Acute care hospital: Home		

References

1. Allen, K., Hazelett, S. E., Jarjoura, D., Wright, K., Fosnight, S. M., Kropp, D. J., Hua, K., & Pfister, E. W. (2011). The after discharge care management of low income frail elderly (ad-life) randomized trial: Theoretical framework and study design. *Population Health Management, 14*(3), 137–142. https://doi.org/10.1089/pop.2010.0016

2. Allen, K., Hazelett, S., Jarjoura, D., Wickstrom, G. C., Hua, K., Weinhardt, J., & Wright, K. (2002). Effectiveness of a postdischarge care management model for stroke and transient ischemic attack: A randomized trial. *Journal of Stroke and Cerebrovascular Diseases, 11(*2), 88–98. https://doi.org/10.1053/jscd.2002.127106

3. Allen, K., Hazelett, S. S., Jarjoura, D. D., Hua, K. K., Wright, K. K., Weinhardt, J. J., & Kropp, D. D. (2009). A randomized trial testing the superiority of a post-discharge care management model for stroke survivors. *Journal of Stroke and Cerebrovascular Diseases, 18*(6), 443–452. https://doi.org/10.1016/j.jstrokecerebrovasdis.2009.02.002

4. Allen, J., Ottmann, G., & Roberts, G. (2013). Multi-professional communication for older people in transitional care: A review of the literature. *International Journal of Older People Nursing, 8*(4), 253–269. https://doi.org/10.1111/j.1748-3743.2012.00314.x

5. Arbaje, A., Maron, D. D., Yu, Q., Wendel, V. I., Tanner, E., Boult, C., Eubank, K. J., & Durso, S. C. (2010). The geriatric floating interdisciplinary transition team. *Journal of the American Geriatrics Society, 58*(2), 364–370. https://doi.org/10.1111/j.1532-5415.2009.02682.x

6. Arbaje, A., Kansagara, D. L., Salanitro, A. H., Englander, H. L., Kripalani, S., Jencks, S. F., & Lindquist, L. A. (2014). Regardless of age: Incorporating principles from geriatric medicine to improve care transitions for patients with complex needs. *Journal of General Internal Medicine : JGIM, 29*(6), 932–939. https://doi.org/10.1007/s11606-013-2729-1

7. Avlund, K., Jepsen, E., Vass, M., & Lundemark, H. (2002). Effects of Comprehensive Follow-up Home Visits after Hospitalization on Functional Ability and Readmissions among Old Patients. A Randomized Controlled Study. *Scandinavian Journal of Occupational Therapy, 9*(1), 17–22. https://doi.org/10.1080/110381202753505827

8. Baillie, L., Gallini, A., Corser, R., Elworthy, G., Scotcher, A., & Barrand, A. (2014). Care transitions for frail, older people from acute hospital wards within an integrated healthcare system in England: a qualitative case study. *International Journal of Integrated Care, 14*(1), e009–e009. https://doi.org/10.5334/ijic.1175

9. Berglund, H., Hasson, H., Kjellgren, K., & Wilhelmson, K. (2015). Effects of a continuum of care intervention on frail older persons' life satisfaction: a randomized controlled study. *Journal of Clinical Nursing, 24*(7-8), 1079–1090. https://doi.org/10.1111/jocn.12699

12. Bradway, C., Bixby, M. B., Hirschman, K. B., McCauley, K., & Naylor, M. D. (2013). Case study: Transitional care for a patient with benign prostatic hyperplasia and recurrent urinary tract infections. *Urologic Nursing, 33*(4), 177–200. https://doi.org/10.7257/1053-816x.2013.33.4.177

13. Broderick, J.P. & Abir, M. (2015). Transitions of Care for Stroke Patients: Opportunities to Improve Outcomes. *Circulation Cardiovascular Quality and Outcomes, 8*(6 Suppl 3), S190–S192. https://doi.org/10.1161/CIRCOUTCOMES.115.002288B

14. Bull, M.J. & Roberts, J. (2001). Components of a proper hospital discharge for elders. *Journal of Advanced Nursing, 35*(4), 571–581. https://doi.org/10.1046/j.1365-2648.2001.01873.x

15. Glasby, J., Martin, G., & Regen, E. (2008). Older people and the relationship between hospital services and intermediate care: Results from a national evaluation. *Journal of Interprofessional Care, 22*(6), 639–649. https://doi.org/10.1080/13561820802309729

16. Cameron, S., McKenzie, F., Warnock, L., & Farquhar, D. (2000). Impact of a nurse led multidisciplinary team on an acute medical admissions unit. *Health Bulletin, 58*(6), 512-514.

17. Cavalier, D. & Sickels, L. P. (2015). The fundamentals of reducing HF readmissions. *Nursing Management, 46*(11), 16–22. https://doi.org/10.1097/01.NUMA.0000472762.57989.79

18. Centeno, M. & Kahveci, K. L. (2014). Transitional care models: preventing readmissions for high-risk patient populations. *Critical Care Nursing Clinics of North America, 26*(4), 589–597. https://doi.org/10.1016/j.ccell.2014.08.009

19. Donaho, E.K., Hall, A. C., Gass, J. A., Elayda, M. A., Lee, V.-V., Paire, S., & Meyers, D. E. (2015). Protocol-driven allied health post-discharge transition clinic to reduce hospital readmissions in heart failure. *Journal of the American Heart Association, 4(*12). https://doi.org/10.1161/JAHA.115.002296

20. Duner, A. (2013). Care planning and decision-making in teams in Swedish elderly care: A study of interprofessional collaboration and professional boundaries*. Journal of Interprofessional Care, 27*(3), 246-253. https://doi.org/10.3109/13561820.2012.757730

21. Ebrahimi, Z., Eklund, K., Dahlin-Ivanoff, S., Jakobsson, A., & Wilhelmson, K. (2017). Effects of a continuum of care intervention on frail elders' self-rated health, experiences of security/safety and symptoms: A randomised controlled trial. *Nordic Journal of Nursing Research, 37*(1), 33–43. https://doi.org/10.1177/2057158516668710

22. Eklund, K., Wilhelmson, K., Gustafsson, H., Landahl, S., & Dahlin-Ivanoff, S. (2013). One-year outcome of frailty indicators and activities of daily living following the randomised controlled trial: "Continuum of care for frail older people." *BMC Geriatrics, 13*(1), 76–10 pages. https://doi.org/10.1186/1471-2318-13-76

23. Ekelund, C. & Eklund, K. (2015). Longitudinal effects on self-determination in the RCT "Continuum of care for frail elderly people." *Quality in Ageing, 16*(3), 165–. https://doi.org/10.1108/QAOA-12-2014-0045

24. Farhat, N., Vordenberg, S. E., Marshall, V. D., Suh, T. T., & Remington, T. L. (2019). Evolution of interdisciplinary geriatric transitions of care on readmission rates. *The American Journal of Managed Care, 25*(7), e219–e223.

25. Figved, W., Myrstad, M., Saltvedt, I., Finjarn, M., Flaten Odland, L. M., & Frihagen, F. (2019). Team Approach: Multidisciplinary Treatment of Hip Fractures in Elderly Patients: Orthogeriatric Care. *JBJS Reviews*, 7(6), e6–e6. https://doi.org/10.2106/JBJS.RVW.18.00136

26. Fjærtoft, Indredavik, B., Johnsen, R., & Lydersen, S. (2004). Acute stroke unit care combined with early supported discharge. Long-term effects on quality of life. A randomized controlled trial. Clinical Rehabilitation, 18(5), 580–586. https://doi.org/10.1191/0269215504cr773oa

27. Fjærtoft, H., Indredavik, B., & Lydersen, S. (2003). Stroke unit care combined with early supported discharge: long-term follow-up of a randomized controlled trial. *Stroke, 34*(11), 2687-2691. https://doi.org/10.1161/01.STR.0000095189.21659.4F

28. Garcia-Aymerich, J., Hernandez, C., Alonso, A., Casas, A., Rodriguez-Roisin, R., Anto, J. M., & Roca, J. (2007). Effects of an integrated care intervention on risk factors of COPD readmission*. Respiratory Medicine, 101*(7), 1462–1469. https://doi.org/10.1016/j.rmed.2007.01.012

29. Garnier, A., Rouiller, N., Gachoud, D., Nachar, C., Voirol, P., Griesser, A., Uhlmann, M., Waeber, G., & Lamy, O. (2018). Effectiveness of a transition plan at discharge of patients hospitalized with heart failure: a before-and-after study. *ESC Heart Failure, 5*(4), 657–667. https://doi.org/10.1002/ehf2.12295

30. Goldstein, & Goldfarb, D. S. (2017). Meeting the Needs of the Complex Older Adult Patient with Urinary Retention: A Case Study. Urologic Nursing, 37(2), 75–100. https://doi.org/10.7257/1053-816X.2017.37.2.75

31. Gray, L., Travers, C. M., Bartlett, H. P., Crotty, M., & Cameron, I. D. (2008). Transition Care: will it deliver? *Medical Journal of Australia, 188*(4), 251–253. https://doi.org/10.5694/j.1326-5377.2008.tb01600.x

34. Hasson, H., Blomberg, S., & Dunér, A. (2012). Fidelity and moderating factors in complex interventions: a case study of a continuum of care program for frail elderly people in health and social care. *Implementation Science,* 7(1), 23–23. https://doi.org/10.1186/1748-5908-7-23

35. Hazelett, S. (2006). after discharge management of low-income frail elderly (AD-LIFE). ClinicalTrials.gov

36. Hennessey, B. & Suter, P. (2011). The Community-Based Transitions Model: one agency's experience. Home Healthcare Nurse, 29(4), 218–230. https://doi.org/10.1097/NHH.0b013e318211986d

38. Hofstad, H., Gjelsvik, B. E. B., Næss, H., Eide, G. E., & Skouen, J. S. (2014). Early supported discharge after stroke in Bergen (ESD Stroke Bergen): three and six months results of a randomized controlled trial comparing two early supported discharge schemes with treatment as usual. *BMC Neurology, 14*(1), 239–239. https://doi.org/10.1186/s12883-014-0239-3

39. Hoover, C., Plamann, J., & Beckel, J. (2017). Outcomes of an interdisciplinary transitional care quality improvement project on self-management and health care use in patients with heart failure*. Journal of Gerontological Nursing, 43*(1), 23-31.

40. Indredavik, B., Fjaertoft, H., Ekeberg, G., Løge, A. D., & March, B. (2000). Benefit of an extended stroke unit service with early supported discharge : A randomized, controlled trial. *Stroke* (1970), 31(12), 2989–2994. https://doi.org/10.1161/01.STR.31.12.2989

41. Inglis, S., Pearson, S., Treen, S., Gallasch, T., Horowitz, J. D., & Stewart, S. (2006). Extending the horizon in chronic heart failure: Effects of multidisciplinary, home-based intervention relative to usual care. *Circulation, 114*(23), 2466–2473. https://doi.org/10.1161/CIRCULATIONAHA.106.638122 Inglis et al. (2006)

42. Johansson, B. C. (2012). Care coordination: Key to providing timely treatment, rehabilitation for elderly, at-risk patients. Caring: National Association for Home Care Magazine, 31(1), 40-44.

43. James, L. (2008). All pulling together: the value of combining health and social care. *Nursing Older People, 20*(9), 16–17. https://doi.org/10.7748/nop.20.9.16.s19 James (2008)

44. Karlsson, A., Berggren, M., Gustafson, Y., Olofsson, B., Lindelöf, N., & Stenvall, M. (2016). Effects of geriatric interdisciplinary home rehabilitation on walking ability and length of hospital stay after hip fracture: a randomized controlled trial. *Journal of the American Medical Directors Association, 17*(5), 464.e9–464.e15. https://doi.org/10.1016/j.jamda.2016.02.001

45. Kongensgaard, R., Hansen, T. K., Krogseth, M., & Gregersen, M. (2022). Impact of involvement of relatives in early home visits by a hospital-led geriatric team. Geriatric Nursing (New York), 45, 64–68. https://doi.org/10.1016/j.gerinurse.2022.02.027

46. Lainscak,M., Kadivec, S., Kosnik, M., Benedik, B., Bratkovic, M., Jakhel, T., Marcun, R., Miklosa, P., Stalc, B., & Farkas, J. (2013). Discharge coordinator intervention prevents hospitalizations in patients with copd: a randomized controlled trial. *Journal of the American Medical Directors Association, 14*(6), 450.e1–450.e6. https://doi.org/10.1016/j.jamda.2013.03.003

47. Liu, V. & Garwood, C. L. (2015). Medication reconciliation to facilitate transitions of care after hospitalization. American Journal of Health-System Pharmacy, 72(9), 690–693. https://doi.org/10.2146/ajhp140133

48. Ljungberg, C., Hanson, E., & Lovgren, M. (2001). A home rehabilitation program for stroke patients. A pilot study. Scandinavian Journal of Caring Sciences, 15(1), 44–53. https://doi.org/10.1046/j.1471-6712.2001.1510044.x

49. Lovelace, D., Hancock, D., Hughes, S. S., Wyche, P. R., Jenkins, C., & Logan, C. (2016). A Patient-centered transitional care case management program: taking case management to the streets and beyond. *Professional Case Management, 21*(6), 277–290. https://doi.org/10.1097/NCM.0000000000000158

50. Low, L. L., Tan, S. Y., Ng, M. J. M., Tay, W. Y., Ng, L. B., Balasubramaniam, K., Towle, R. M., & Lee, K. H. (2017). Applying the integrated practice unit concept to a modified virtual ward model of care for patients at highest risk of readmission: A randomized controlled trial. *PloS One, 12*(1), e0168757–e0168757. https://doi.org/10.1371/journal.pone.0168757

51. Low, L. L., Tan, A. K. H., & Vasanwala, F. F. (2014). Collaboration between two restructured hospitals' family physician-led transitional home care teams in the provision of home ventilation respiratory support. *Proceedings of Singapore Healthcare, 23*(2), 173–176. https://doi.org/10.1177/201010581402300213

52. Lowthian, J. (2017). How do we optimise care transition of frail older people? Age and Ageing, 46(1), 2–4. https://doi.org/10.1093/ageing/afw171

53. Mashaw, A. (2014). implementation of a hospital readmissions prevention program in a rural geriatric population. *Journal of the American Geriatrics Society (JAGS), 62*(10), 1998–1999. https://doi.org/10.1111/jgs.13053

54. Manley, H., Aweh, G., Weiner, D. E., Jiang, H., Miskulin, D. C., Johnson, D., & Lacson, E. K. (2020). Multidisciplinary medication therapy management and hospital readmission in patients undergoing maintenance dialysis: A retrospective cohort study. American Journal of Kidney Diseases, 76(1), 13–21. https://doi.org/10.1053/j.ajkd.2019.12.002

55. Markle-Reid, M., Valaitis, R., Bartholomew, A., Fisher, K., Fleck, R., Ploeg, J., Salerno Aging, J., Thabane, L., Gafni, A., & Archer, N. (2019). Implementation and evaluation of an integrated hospital-to-home transitional care intervention for older adults with stroke and multimorbidity: A feasibility study. *International Journal of Integrated Care, 19*(4), 367–. https://doi.org/10.5334/ijic.s3367

56. Miranda, M., Gorski, L. A., LeFevre, J. G., Levac, K. A., Niederstadt, J. A., & Toy, A. L. (2002). An evidence-based approach to improving care of patients with heart failure across the continuum. *Journal of Nursing Care Quality, 17*(1), 1–14. https://doi.org/10.1097/00001786-200210000-00002

57. Mudge, A., Barras, M., Adsett, J., Mullins, R. W., Lloyd, S., & Kasper, K. (2014). improving care transitions in individuals frequently admitted to the hospital. *Journal of the American Geriatrics Society (JAGS), 62*(10), 1994–1996. https://doi.org/10.1111/jgs.13033

58. Naylor, & Sochalski, J. A. (2010). Scaling up: Bringing the transitional care model into the mainstream. *Issue Brief (Commonwealth Fund), 103*, 1–12.

59. Naylor, M., Hirschman, K. B., & McCauley, K. (2020). Meeting the transitional care needs of older adults with COVID-19. Journal of Aging & Social Policy, 32(4-5), 387–395. https://doi.org/10.1080/08959420.2020.1773189

60. Oboh, L. (2016). Communication during transfer of care of older people. *The Pharmaceutical Journal, 296*(10.1211).

61. Ohuabunwa, U., Jordan, Q., Shah, S., Fost, M., & Flacker, J. (2013). Implementation of a care transitions model for low-income older adults: A high-risk, vulnerable population. Journal of the American Geriatrics Society (JAGS), 61(6), 987–992. https://doi.org/10.1111/jgs.12276

62. Ornstein, K., Smith, K. L., Foer, D. H., Lopez-Cantor, M. T., & Soriano, T. (2011). To the hospital and back home again: a nurse practitioner-based transitional care program for hospitalized homebound people. *Journal of the American Geriatrics Society (JAGS), 59*(3), 544–551. https://doi.org/10.1111/j.1532-5415.2010.03308.x

63. Palmer, R. (2018). The acute care for elders unit model of care. *Geriatrics (Basel), 3*(3), 59–. https://doi.org/10.3390/geriatrics3030059

64. Ponce de Leon, M. & Hohler, A. D. (2017). Safety Considerations During Transitions of Care From Inpatient to Outpatient Settings. Continuum (Minneapolis, Minn.), 23(3, Neurology of Systemic Disease), 877–881. https://doi.org/10.1212/con.0000000000000465

65. Preen, Bailey, B. E. S., Wright, A., Kendall, P., Phillips, M., Hung, J., Hendriks, R., Mather, A., & Williams, E. (2005). Effects of a multidisciplinary, post-discharge continuance of care intervention on quality of life, discharge satisfaction, and hospital length of stay: A randomized controlled trial. International Journal for Quality in Health Care, 17(1), 43–51. https://doi.org/10.1093/intqhc/mzi002

66. Reinhard, S. C., & Lind, K. D. (2011). Public Policy Implications for Pathways Through Transitions. In Charting the Future on Transitions of Care. Annual Review of Gerontology and Geriatrics. Doi.org.10/1891/0198-8794.31.209

67. Schapira, M., Outumuro, M. B., Giber, F., Pino, C., Mattiussi, M., Montero-Odasso, M., Boietti, B., Saimovici, J., Gallo, C., Hornstein, L., Pollán, J., Garfi, L., Osman, A., & Perman, G. (2022). Geriatric co-management and interdisciplinary transitional care reduced hospital readmissions in frail older patients in Argentina: results from a randomized controlled trial. *Aging Clinical and Experimental Research, 34*(1), 85–93. https://doi.org/10.1007/s40520-021-01893-

68. Sheikh, F., Gathecha, E., Bellantoni, M., Christmas, C., Lafreniere, J. P., & Arbaje, A. I. (2018). A call to bridge across silos during care transitions*. Joint Commission Journal on Quality And Patient Safety*, 44(5), 270–278. https://doi.org/10.1016/j.jcjq.2017.10.006

69. Sims-Gould, J., Byrne, K., Hicks, E., Khan, K., & Stolee, P. (2012). Examining “success” in post-hip fracture care transitions: A strengths-based approach. Journal of Interprofessional Care, 26(3), 205–211. https://doi.org/10.3109/13561820.2011.645090

70. Stranges, P., Marshall, V. D., Walker, P. C., Hall, K. E., Griffith, D. K., & Remington, T. (2015). A multidisciplinary intervention for reducing readmissions among older adults in a patient-centered medical home. *The American Journal of Managed Care, 21*(2), 106–113.

71. Tahan, H. (2007). One patient, numerous healthcare providers, and multiple care settings: addressing the concerns of care transitions through case management. *Professional Case Management*, *12*(1), 37–46. https://doi.org/10.1097/01269241-200701000-00008 Tahan (2007)

72. Takahashi, P., Leppin, A. L., & Hanson, G. J. (2020). hospital to community transitions for older adults: an update for the practicing clinician. *Mayo Clinic Proceedings, 95*(10), 2253–2262. https://doi.org/10.1016/j.mayocp.2020.02.001

73. Thorne, D., & Jeffery, S. (2001). Intermediate care. Homeward bound. The Health Service Journal, 111(5785), 28-29.

74. Torp, Vinkler, S., Pedersen, K. D., Hansen, F. R., Jørgensen, T., & Olsen, J. (2006). Model of hospital-supported discharge after stroke. *Stroke* (1970), 37(6), 1514–1520. https://doi.org/10.1161/01.STR.0000221793.81260.ed

75. Verweij, L., Jepma, P., Buurman, B. M., Latour, C. H. M., Engelbert, R. H. H., ter Riet, G., Karapinar-Çarkit, F., Daliri, S., Peters, R. J. G., & Scholte op Reimer, W. J. M. (2018). The cardiac care bridge program: Design of a randomized trial of nurse-coordinated transitional care in older hospitalized cardiac patients at high risk of readmission and mortality. *BMC Health Services Research, 18*(1), 508–508. https://doi.org/10.1186/s12913-018-3301-9

76. Watson, A., Charlesworth, L., Jacob, R., Kendrick, D., Logan, P., Marshall, F., Montgomery, A., Sach, T., Tan, W., Walker, M., Waring, J., Whitham, D., & Sahota, O. (2015). The Community In-Reach and Care Transition (CIRACT) clinical and cost-effectiveness study: Study protocol for a randomised controlled trial. *Trials, 16*(1), 41–41. https://doi.org/10.1186/s13063-015-0551-2)

77. Weintraub, W., Elliott, D., Fanari, Z., Ostertag-Stretch, J., Muther, A., Lynahan, M., Kerzner, R., Salam, T., Scherrer, H., Anderson, S., Russo, C. A., Kolm, P., & Steinberg, T. H. (2018). The impact of care management information technology model on quality of care after coronary artery bypass surgery: “Bridging the divides.” *Cardiovascular Revascularization Medicine, 19*(1), 106–111. https://doi.org/10.1016/j.carrev.2017.06.008

78. Wierzchowiecki, M., Poprawski, K., Nowicka, A., Kandziora, M., Piątkowska, A., Jankowiak, M., ... & Michalski, M. (2006). Original article A new programme of multidisciplinary care for patients with heart failure in Poznań: One-year follow-up. Kardiologia Polska (Polish Heart Journal), 64(10), 1063-1070.

79. Winfield, A. & Burns, E. (2016). Let’s all get home safely: A commentary on NICE and SCIE guidelines (NG27) transition between inpatient hospital settings and community or care home settings. Age and Ageing, 45(6), 757–760. https://doi.org/10.1093/ageing/afw151

80. Wright, K., Hazelett, S., Jarjoura, D., & Allen, K. (2007). The AD-LIFE trial: working to integrate medical and psychosocial care management models. Home Healthcare Nurse, 25(5), 308–314. https://doi.org/10.1097/01.NHH.0000269964.34045.d5
