# Supplementary material for: Interprofessional Teams Supporting Care Transitions from Hospital to Community: A Scoping Review
Source: Int J Integr Care. 2024 Apr 2;24(2):1. doi: 10.5334/ijic.7623 (PMC11012160; doi:10.5334/ijic.7623)
Supplement: Appendix C. — Interventions/Programs in the Body of Literature. [file ijic-24-2-7623-s3.pdf]

Appendix C: **Interventions/Programs in the Body of Literature** ***with Bridging Models in Italics***

1. Post discharge care management model for stroke and TIA (1)
2. After Discharge Management of Low Income Frail Elderly (AD-LIFE) trial (AD Life) (2-4)
3. Geriatric Floating Interdisciplinary Transition Team (Geri-FITT) (5)
4. Comprehensive follow-up visits(6)
5. Continuum of care intervention (7-11)
6. Nurse-led multidisciplinary team (12)
7. Heart failure collaborative team (13)
8. Transitional care model (14)
9. Interdisciplinary transitions of care service (15)
10. Extended stroke unit service (ESUS) (16-18)
11. Integrated care (IC) intervention (19)
12. Multidisciplinary transition plan for HF (20)
13. Early supported discharge (21)
14. Coleman intervention for heart failure (22)
15. Home-based intervention (HBI) (23)
16. Geriatric Interdisciplinary Home Rehabilitation (GIHR) (24)
17. Early home visits by hospital geriatric team (25)
18. Discharge coordinator intervention for COPD (26)
19. Multidisciplinary transitions of care team (27)
20. Veterans Administration Medical Center (VAMC) transitional care program (TCP) (28)
21. Modified Virtual Ward Care (29)
22. Multidisciplinary medication therapy management (MTM) (30)
23. Readmission prevention team (31)
24. Multidisciplinary follow-up clinic (32)
25. Coleman intervention for low income (33)
26. Nurse practitioner transitional care program (34)

27. Acute Care for Elderly (ACE) (35)
28. Discharge care planning model (36)
29. Geriatric co-management model (37)
30. Patient-centred medical home intervention (38)
31. Area-wide stroke strategy (39)
32. Hospital-supported discharge after stroke (40)
33. Cardiac Care Bridge (41)
34. Community in-reach and care transition service (42)
35. Multidisciplinary care for heart failure (43)

References

1. Allen KR, Hazelett S, Jarjoura D, Wickstrom GC, Hua K, Weinhardt J, et al. Effectiveness of a postdischarge care management model for stroke and transient ischemic attack: a randomized trial. Journal of stroke and cerebrovascular diseases. 2002;11(2):88-98.
2. Allen KR, Hazelett SE, Jarjoura D, Wright K, Fosnight SM, Kropp DJ, et al. The after Discharge Care Management of Low Income Frail Elderly (AD-LIFE) randomized trial: Theoretical framework and study design. Population Health Management. 2011;14(3):137-42.
3. Hazelett S. After discharge management of low income frail elderly (AD-LIFE). ClinicalTrialsgov [Internet]. 2006.
4. Wright K, Hazelett S, Jarjoura D, Allen K. The AD-LIFE trial: Working to integrate medical and psychosocial care management models. Home Healthcare Nurse. 2007;25(5):308-14.
5. Arbaje AI, Maron DD, Yu Q, Wendel VI, Tanner E, Boult C, et al. The geriatric floating interdisciplinary transition team. J Am Geriatr Soc. 2010;58(2):364-70.
6. Avlund K, Jepsen E, Vass M, Lundemark H. Effects of comprehensive follow-up home visits after hospitalization on functional ability and readmissions among old patients. A randomized controlled study. Scandinavian Journal of Occupational Therapy. 2002;9(1):17-22.
7. Berglund H, Hasson H, Kjellgren K, Wilhelmson K. Effects of a continuum of care intervention on frail older persons' life satisfaction: a randomized controlled study. Journal of Clinical Nursing (John Wiley & Sons, Inc). 2015;24(7):1079-90.
8. Ebrahimi Z, Eklund K, Dahlin-Ivanoff S, Jakobsson A, Wilhelmson K. Effects of a continuum of care intervention on frail elders' self-rated health, experiences of security/safety and symptoms: A randomised controlled trial. Nordic Journal of Nursing Research. 2017;37(1):33-43.
9. Ekelund C, Eklund K. Longitudinal effects on self-determination in the RCT "Continuum of care for frail elderly people". Quality in Ageing & Older Adults. 2015;16(3):165-76.
10. Eklund K, Wilhelmson K, Gustafsson H, Landahl S, Dahlin-Ivanoff S. One-year outcome of frailty indicators and activities of daily living following the randomised controlled trial: "Continuum of care for frail older people". BMC Geriatr. 2013;13:76.

11. Hasson H, Blomberg S, Duner A. Fidelity and moderating factors in complex interventions: a case study of a continuum of care program for frail elderly people in health and social care. Implementation science : IS. 2012;7:23.
12. Cameron S, McKenzie F, Warnock L, Farquhar D. Impact of a nurse led multidisciplinary team on an acute medical admissions unit. Health bulletin. 2000;58(6):512-4.
13. Cavalier DK, Sickels LP. The fundamentals of reducing HF readmissions. Nursing Management. 2015;46(11):16-22.
14. Centeno MM, Kahveci KL. Transitional Care Models. Critical Care Nursing Clinics of North America. 2014;26(4):589-97.
15. Farhat NM, Vordenberg SE, Marshall VD, Suh TT, Remington TL. Evolution of interdisciplinary geriatric transitions of care on readmission rates. The American journal of managed care. 2019;25(7):e219-e23.
16. Fjaertoft H, Indredavik B, Lydersen S. Stroke unit care combined with early supported discharge: long-term follow-up of a randomized controlled trial. Stroke. 2003;34(11):2687-91.
17. Fjaertoft H, Indredavik B, Johnsen R, Lydersen S. Acute stroke unit care combined with early supported discharge. Long-term effects on quality of life. A randomized controlled trial. Clinical rehabilitation. 2004;18(5):580-6.
18. Indredavik B, Fjaertoft H, Ekeberg G, Løge AD, Mørch B. Benefit of an extended stroke unit service with early supported discharge: a randomized, controlled trial. Stroke; a journal of cerebral circulation. 2000;31(12):2989-94.
19. Garcia-Aymerich J, Hernandez C, Alonso A, Casas A, Rodriguez-Roisin R, Anto JM, et al. Effects of an integrated care intervention on risk factors of COPD readmission. Respiratory Medicine. 2007;101(7):1462-9.
20. Garnier A, Rouiller N, Gachoud D, Nachar C, Voirol P, Griesser AC, et al. Effectiveness of a transition plan at discharge of patients hospitalized with heart failure: a before-and-after study. ESC Heart Fail. 2018;5(4):657-67.
21. Hofstad H. Scandinavian challenges in geriatric rehabilitation: early discharge for stroke patients. European geriatric medicine. 2014;5:S8-S9.
22. Hoover C, Plamann J, Beckel J. Outcomes of an Interdisciplinary Transitional Care Quality Improvement Project on Self-Management and Health Care Use in Patients With Heart Failure. Journal of Gerontological Nursing. 2017;43(1):23-31.
23. Inglis SC, Pearson S, Treen S, Gallasch T, Horowitz JD, Stewart S. Extending the horizon in chronic heart failure: effects of multidisciplinary, home-based intervention relative to usual care. Circulation. 2006;114(23):2466-73.
24. Karlsson A, Berggren M, Gustafson Y, Olofsson B, Lindelof N, Stenvall M. Effects of Geriatric Interdisciplinary Home Rehabilitation on Walking Ability and Length of Hospital Stay After Hip Fracture: a Randomized Controlled Trial. Journal of the american medical directors association. 2016;17(5):464.e9-.e15.
25. Kongensgaard R, Hansen, T. K., Krogseth, M., & Gregersen, M. Impact of involvement of relatives in early home visits by a hospital-led geriatric team Geriatric Nursing (New York). 2022;45:64-8.
26. Lainscak M, Kadivec S, Kosnik M, Benedik B, Bratkovic M, Jakhel T, et al. Discharge coordinator intervention prevents hospitalizations in patients with COPD: A randomized controlled trial. Journal of the American Medical Directors Association. 2013;14(6):450.
27. Liu VC, Garwood CL. Medication reconciliation to facilitate transitions of care after hospitalization. American Journal of Health-System Pharmacy. 2015;72(9):690-3.

28. Lovelace D, Hancock D, Hughes SS, Wyche PR, Jenkins C, Logan C. A Patient-Centered Transitional Care Case Management Program. Professional Case Management. 2016;21(6):277-90.
29. Low LL, Tan SY, Ng MJ, Tay WY, Ng LB, Balasubramaniam K, et al. Applying the Integrated Practice Unit Concept to a Modified Virtual Ward Model of Care for Patients at Highest Risk of Readmission: a Randomized Controlled Trial. PloS one. 2017;12(1):e0168757.
30. Manley HJ, Gideon Aweh, Daniel E. Weiner, Huan Jiang, Dana C. Miskulin, Doug Johnson, and Eduardo K. Lacson. . Multidisciplinary medication therapy management and hospital readmission in patients undergoing maintenance dialysis: a retrospective cohort study American Journal of Kidney Diseases. 2020;76(1):13-21.
31. Mashaw A. Implementation of a hospital readmissions prevention program in a rural geriatric population. Journal of the American Geriatrics Society. 2014;62(10):1998-9.
32. Mudge AM, Barras M, Adsett J, Mullins RW, Lloyd S, Kasper K. Improving care transitions in individuals frequently admitted to the hospital. Journal of the American Geriatrics Society. 2014;62(10):1994-6.
33. Ohuabunwa U, Jordan Q, Shah S, Fost M, Flacker J. Implementation of a care transitions model for low-income older adults: a high-risk, vulnerable population. J Am Geriatr Soc. 2013;61(6):987-92.
34. Ornstein K, Smith KL, Foer DH, Lopez-Cantor MT, Soriano T. To the hospital and back home again: a nurse practitioner-based transitional care program for hospitalized homebound people. J Am Geriatr Soc. 2011;59(3):544-51.
35. Palmer RM. The Acute Care for Elders Unit Model of Care. Geriatrics (Basel, Switzerland). 2018;3(3).
36. Preen DB, Bailey BES, Wright A, Kendall P, Phillips M, Hung J, et al. Effects of a multidisciplinary, post-discharge continuance of care intervention on quality of life, discharge satisfaction, and hospital length of stay: a randomized controlled trial. International journal for quality in health care : journal of the International Society for Quality in Health Care. 2005;17(1):43-51.
37. Schapira M, Camera L, Finkelsztein C, Matusevich D, Smietniansky M, Esteban J, et al. A multidisciplinary program for the treatment and follow up of depression in ambulatory elderly. Ensayo pragmatico para evaluar la efectividad de un programa de manejo de depresion para pacientes mayores de 65 anos en un Plan de Salud. 2010;21(92):284-90.
38. Stranges PM, Marshall VD, Walker PC, Hall KE, Griffith DK, Remington T. A multidisciplinary intervention for reducing readmissions among older adults in a patient-centered medical home. The American journal of managed care. 2015;21(2):106-13.
39. Thorne D, Jeffery S. Intermediate care. Homeward bound. The Health service journal. 2001;111(5785):28-9.
40. Torp CR, Vinkler S, Pedersen KD, Hansen FR, Jorgensen T, Willaing I, et al. Model of hospital-supported discharge after stroke. Stroke. 2006;37(6):1514-20.
41. Verweij L, Jepma P, Buurman BM, Latour CHM, Engelbert RHH, Ter Riet G, et al. The cardiac care bridge program: design of a randomized trial of nurse-coordinated transitional care in older hospitalized cardiac patients at high risk of readmission and mortality. BMC health services research. 2018;18(1):508.
42. Watson A, Charlesworth L, Jacob R, Kendrick D, Logan P, Marshall F, et al. The Community In-Reach and Care Transition (CIRACT) clinical and cost-effectiveness study: Study protocol for a randomised controlled trial. Trials. 2015;16(1):41.

43. Wierzchowiecki M, Poprawski K, Nowicka A, Kandziora M, Piatkowska A, Jankowiak M, et al. A new programme of multidisciplinary care for patients with heart failure in Poznań: one-year follow-up. Kardiologia polska. 2006;64(10):1063-70; discussion 71-2.
